# Adolescent/Youth Reproductive Mobile Access and Delivery Initiative for Love and Life Outcomes (ARMADILLO) Study: formative protocol for mHealth platform development and piloting

**DOI:** 10.1186/s12978-015-0059-y

**Published:** 2015-08-07

**Authors:** Lianne Gonsalves, Kelly L. L’Engle, Tigest Tamrat, Kate F. Plourde, Emily R. Mangone, Smisha Agarwal, Lale Say, Michelle J. Hindin

**Affiliations:** Department of Reproductive Health and Research including UNDP/UNFPA/UNICEF/WHO/World Bank Special Programme of Research, Development and Research Training in Human Reproduction (HRP), World Health Organization, Avenue Appia 20, 1201 Geneva, Switzerland; FHI360, 359 Blackwell Street, Suite 200, Durham, NC 27701 USA; Health Policy & Management, UNC Gillings School of Global Public Health, University of North Carolina, 135 Dauer Drive, Chapel Hill, NC 27599-7400 USA; Department of Maternal and Child Health, UNC Gillings School of Global Public Health, University of North Carolina, 135 Dauer Drive, Chapel Hill, NC 27599-7400 USA

**Keywords:** Adolescent, Youth, mHealth, Intervention, Low-income countries, Middle-income countries

## Abstract

**Background:**

There is a high unmet need for sexual and reproductive health (SRH) information and services among youth (ages 15-24) worldwide (MacQuarrie KLD. Unmet Need for Family Planning among Young Women: Levels and Trends 2014). With the proliferation of mobile technology, and its popularity with this age group, mobile phones offer a novel and accessible platform for a discreet, on-demand service providing SRH information. The Adolescent/Youth Reproductive Mobile Access and Delivery Initiative for Love and Life Outcomes (ARMADILLO) formative study will inform the development of an intervention, which will use the popular channel of SMS (text messages) to deliver SRH information on-demand to youth.

**Methods/Design:**

Following the development of potential SMS message content in partnership with SRH technical experts and youth, formative research activities will take place over two phases. Phase 1 will use focus group discussions (FGDs) with youth and parents/caregivers to develop and test the appropriateness and acceptability of the SMS messages. Phase 2 will consist of ‘peer piloting’, where youth participants will complete an SRH outcome-focused pretest, be introduced to the system and then have three weeks to interact with the system and share it with friends. Participants will then return to complete the SRH post-test and participate in an in-depth interview about their own and their peers’ opinions and experiences using ARMADILLO.

**Discussion:**

The ARMADILLO formative stage will culminate in the finalization of country-specific ARMADILLO messaging. Reach and impact of ARMADILLO will be measured at later stages. We anticipate that the complete ARMADILLO platform will be scalable, with the potential for national-level adoption.

## Background

There are 1.8 billion people between 10 and 24 years of age, 90% of whom live in low- and middle-income countries [1]. Each year, 16 million girls between 15 and 19 years of age and two million girls under 15 years of age give birth. It is estimated that a lack of access to contraception leads to 7.4 million unintended pregnancies among adolescents, aged 10 to 19 [2, 3]. Unintended pregnancies resulted in an estimated 3.2 million unsafe abortions worldwide in 2008, and complications related to pregnancy and childbirth are the leading cause of death for women ages 15-19 [3]. There is a high unmet need for sexual and reproductive health (SRH) services, for both married and unmarried youth worldwide [4]. However, financial, cultural, social, and legal considerations often impede youth access to the sexual and reproductive health resources that may be available in their communities.

Barriers to youth accessing contraception include limited sources of reliable information about sexual and reproductive health; cultural and social stigma surrounding use of contraceptives; provider biases towards treating young or unmarried individuals, and cost and convenience of accessing SRH services when available [5]. While there are efforts in place to make facility-based services and health providers ‘youth-friendly,’ there is a need to strengthen the enabling environment for youth to actually seek care [6]. This includes using channels outside of the facility to reach youth with high-quality SRH content and details about the SRH services that are available to them.

Many strategies have relied on creative uses of mass media to engage and educate youth – radio, magazines, comics, and television have entertained young people while disseminating quality, accurate information on a wide array of sexual and reproductive health and rights issues [7–9]. The proliferation of mobile technology in recent years and its popularity with young people offers another modality to reach this age group. Of the nearly 6 billion mobile phone subscribers worldwide, approximately one-third are under the age of 30 [10]. Given the proliferation of mobile phones, they offer the potential of discretely delivering tailored sexual and reproductive health content without stigma or judgment.

Programs using mobile phones have been used in many areas of health care and health promotion globally [11–15]. Recent reviews of studies using mobile phones to promote behaviour change have demonstrated mixed to positive effects of such programs [16–19]. Studies in the United States, Australia, and South Africa, for example, confirm that text messaging programs for reproductive health can lead to better sexual health and increased use of health services [20, 21]. Text messaging programs are the most common approach to behaviour change using mobile technologies, as texting is one of the most frequent forms of mobile phone communication [22, 23], and is available on every mobile phone regardless of phone model or mobile network provider, with minimal costs per message.

There are numerous projects being implemented globally that leverage mobile technologies for improved youth SRH. FHI360’s m4RH program uses SMS (text messages) to provide information about nine family planning methods as well as a clinic database in Kenya and Tanzania. m4RH adaptations such as mCENAS in Mozambique, have expanded on this strategy and use SMS to provide access to broader SRH information targeted especially for youth. Other examples linking technology to youth-targeted SRH information dissemination include: the YoungAfricaLive mobile portal for discussing love, sex, relationships, gender and cultural issues, as well as HIV/AIDS in South Africa; the school-based Learning about Living program developed around the Nigerian Family Life and HIV/AIDS education; the Access, Service & Knowledge (ASK) programme targeting young people between the ages of 10 and 24 years to disseminate information on SRH and HIV/AIDS in seven countries through mobile and electronic platforms; and various mHealth strategies to engage young people implemented by Marie Stopes International.

Studies in Kenya and Tanzania have highlighted the feasibility of sexual and reproductive health content delivered to young people via mobile phones [24]. Findings suggested that in both countries, a majority of the users accessing this mobile content were under 30 years of age. Participants liked receiving family planning information via mobile phones, as it was confidential and easy-to-understand [25]. Additionally, these studies also suggested improved client-reported family-planning practices and health care-seeking as a result of improved knowledge about contraceptive methods [24]. However, while there is some evidence to suggest high acceptability of mobile phone-based sexual health education among young people, there are minimal data to suggest that such an intervention can result in sustained change in sexual health knowledge, behaviour and practices. Additionally, the evidence on feasibility of mobile-based health behaviour change interventions is still in its infancy, and its application among youth populations needs to be tested in varied settings in order to incorporate appropriate adaptations of such delivery mechanisms among youth populations. The global health community has issued repeated calls for evidence, particularly around the impact of mobile phone approaches on achieving improved health of populations [26, 27].

To answer these calls, the World Health Organization’s Department of Reproductive Health and Research partnered with FHI360 to initiate the ARMADILLO (Adolescent/Youth Reproductive Mobile Access and Delivery Initiative for Love and Life Outcomes) Study, with the goal of developing and evaluating an on-demand system for youth to access and receive SRH information through SMS. In this protocol, we describe the formative phase of this study, which uses qualitative methods to develop and test messages as well as assess the feasibility and usability of ARMADILLO. Following the completion of formative research, subsequent research will assess the impact and coverage of the ARMADILLO platform. ARMADILLO is hypothesized to impact SRH knowledge, norms, and self-efficacy, while also possibly influencing SRH behaviour.

## Methods

### Intervention design

ARMADILLO will consist of an automated, interactive, and on-demand short message service (SMS, also known as ‘text message’) platform that will provide essential facts and address common misconceptions about a full range of SRH issues pertinent to youth, including puberty, sex and pregnancy, HIV and STIs, and contraception. Additionally, the platform will contain various role model stories (featuring fictional peers modelling healthy SRH behaviours, including use of health services). The ARAMDILLO system will be available to users at no charge.

The ARMADILLO platform’s intended use of on-demand access to SRH information delivered via SMS is but one strategy that capitalizes on young people’s widespread use of mobile and electronic devices to improve information on and access to SRH services. Push message services (where users subscribe to a service and are automatically sent messages from the services until opting out), phone hotlines, computer-based interactive teaching programs in schools, and online forums are all strategies which have been used to engage young people around SRH issues. However, ARMADILLO opts to work exclusively with SMS in order to maximize its scalable potential - SMS is a channel for communication available on all mobile phones.

### Study setting

The study will take place in two sites that meet the following criteria:

- a legal framework allowing for youth access to SRH information and services in place;
- demonstrated support for the development and implementation of adolescent or youth-friendly health services;
- demonstrated reproductive health needs among youth populations; and
- accessible mobile infrastructure in place and a high penetration of mobile phones among the population.

### Study design

The formative research phase centres around the development of content for ARMADILLO and piloting of the resulting platform. Formative research activities will include substantial youth participation, and will occur in two phases.

Prior to the start of formative research activities, the local research partner will convene a group of youth sexual and reproductive health (YSRH) stakeholders to inform the development of the ARMADILLO message content. Members might include government technical experts from relevant ministries, representatives from civil society and non-governmental organizations, health providers, and youth health advocates. Their representation in the group would be matched with equal numbers of youth representatives (for example peer educators or youth leaders). This stakeholder group will establish SRH domains and subdomains around which to develop youth-targeted SRH content that is sensitive to local culture and reflective of national policies and guidelines around YSRH services.

Following domain identification, the local research partner will work with the global ARMADILLO team to write draft message content. The message development group of stakeholders will review and prioritize the localized draft content, ensuring key health messages are present and conveyed in a manner appropriate for the cultural context. Translated, locally adapted content, and a platform architecture (with set SRH health topics) that reflects national and local YSRH priorities and guidelines will be the major output of the collaboration between the stakeholder group and local research team prior to the initiation of study activities. Additionally, this group will provide input throughout each of the formative research phases, which are detailed below.

### Phase 1: Formative concept testing

The first phase of the formative research consists of a series of focus group discussions (FGDs) with two sets of participants – youth and parents/caregivers of youth - to test the appropriateness and acceptability of the messages. The FGDs will provide an in-depth understanding of youth mobile phone usage and youth information interests, needs, and concerns related to SRH, which will strengthen the content of ARMADILLO. Youth and adult participants will be able to provide feedback on areas that they would like to see modified. Participants will be shown a prototype of the system as well as proposed promotional materials to assess comprehension, usability, and likelihood of use.

Focus groups will be conducted in a private room, at a time that is most convenient for participants. Each focus group will be conducted with a moderator and note taker. The moderator will be responsible for leading the discussions, probing participants for more information, and asking the next series of questions. Note takers will record the participants’ responses and non-verbal reactions.

All discussions will be digitally recorded. In order to protect privacy, at no point during the study activity will participants be referred to by their name; instead, all participants will be assigned an identifying name/number. Moderators will refer to participants by this name/number in their notes and in the discussion, and participants will be asked to refer to others in the FGD by this same identifier. Additionally, at the start and completion of the FGD, moderators will inform/remind the participants that they should protect the privacy of fellow participants by not sharing information about what was discussed during their participation. During each of the focus group discussions, facilitators will use a discussion guide and interface mock-up - rather than an actual mobile phone - to test ARMADILLO concepts, module content, and message language. Youth and parents/caregivers of youth participants will have the opportunity to provide feedback on the proposed messages to be included by the program and can suggest additional or revised content to be included. At the beginning of the FGD, participants will be informed by moderators that they should not disclose anything personal but should instead refer to third parties (without providing names or other identifying features) or normative practices/opinions. Results from this formative stage will be reviewed and messages modified as dictated by the findings.

### Phase 2: Piloting and finalization

**Fig. 1:**
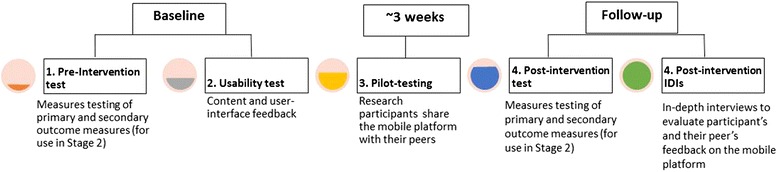
Progression of Phase 2 of the ARMADILLO formative study

In the second phase, youth participants recruited from the target communities will complete a pre-test assessing their baseline knowledge of SRH domain areas covered by ARMADILLO. Then, they will be guided through a usability test by a member of the research team, which will introduce them to the ARMADILLO platform on their mobile phone. Participants will conclude their initial participation with the member of the research team asking them to use ARMADILLO on their mobile phones and show it to five youth peers who are “like them” over a period of three weeks. After three weeks, participants will return to complete an SRH knowledge post-test and participate in an in-depth interview about their own and their peers’ opinions and experiences using ARMADILLO. Phase 2 is detailed further in Fig. 1 below.

Individual interviews and pre-post tests will be held in a private room at each of the sites, during a time that is most convenient for participants. A project staff member will conduct the interview with the participant by reading questions from the interview guide and probing the participant as necessary. All interviews will be digitally recorded.

In addition to testing measures to assess “learnings” from ARMADILLO, the pre-post test will evaluate overall appropriateness and acceptability and inform any final platform content changes. The in-depth interview will provide valuable qualitative data on how ARMADILLO resonates with youth and its effect on interpersonal communications around sensitive SRH topics. Following the pilot, the platform and accompanying promotional plans will be presented as recommendations for the message development group of stakeholders and research team to finalize.

### Study participants

For this formative study, we have identified key participant groups who can contribute important information to intervention development and message testing: youth, parents/caretakers and healthcare providers. We anticipate that the large majority of ARMADILLO users will be 15-24 year olds and thus content and usability testing will be conducted with this age group. It is essential to include youth as participants to understand their perspective on SRH information needs and interests and to provide youth with an opportunity to participate in the development, refinement, and marketing strategy of a tool that is targeted for their age group.

Individuals classified as youth may undergo similar biological changes (puberty) associated with the later stages of adolescence; however the social transitions and shifts in responsibilities vary widely by culture [28]. Therefore, youth study participants will be further divided based on sex and age. This additional stratification by age reflects a sensitivity to the differences in outlook, life, SRH needs, and the transition to adulthood that younger youth may have in comparison to older youth. The age limits for ‘younger’ and ‘older’ youth participants may also vary slightly by study site (for example, 15-17 and 18-24 in one site and 15-19 and 20-24 in a second site), in acknowledgement of diversity in definitions of youth, adulthood and SRH realities for young populations in different countries. Separating younger and older youth will foster an environment where participants feel comfortable actively participating in discussions with their peers, facilitating a more nuanced understanding of the distinct needs of these two groups.

### Young people, Phases 1 and 2

In Phase 1, equal numbers of male and female youths will be recruited for participation in focus group discussions in Phase 1. Focus groups will have a maximum of 10 participants and will be stratified by sex and age. The point of age stratification of participants into ‘younger’ and ‘older’ youth, will be decided by study sites (for reasons explained above), but no participant will be younger than 15 or older than 24. A minimum of 8 and a maximum of 12 FGDs will be conducted with young people.

In Phase 2, equal numbers of male and female and younger and older youth will be recruited to participate in a hands-on, pilot test of the ARMADILLO prototype, with opportunities to use and share the service for approximately three weeks and provide detailed feedback before the system is finalized.

For both phases, eligibility criteria will be as follows:

- Youth between the ages of 15 and 24;
- Regular cell phone users (those who are comfortable using a mobile phone);
- Have a mobile phone with them at the time of recruitment (Phase 2 only);
- Report use of text messaging; and
- Adequate text message health literacy^1^

### Parents/Caregivers, Phase 1

Additionally, two focus groups, one including up to 10 parents and guardians of young people and one including up to 10 teachers, health providers and other youth providers will provide an opportunity to gain their perspectives on SRH issues facing their young people. Based on previous work in similar settings, we have found that one to two focus groups with parents/caregivers provides sufficient insight into youth mobile phone usage and SRH concerns. Our priority is to emphasize first-hand youth perspectives in the development of this intervention while encompassing the broader parent/caregiver perspective. We purposefully include both guardians and caregivers to promote diversity of perspective and conversation generation during the FGD.

Eligibility criteria for participation in this parent focus group will be as follows:

- 18 years or older;
- Regular cell phone users (those who are comfortable using a mobile phone);
- Familiarity with text messaging; and
- Parent, guardian, educator, or SRH service provider of a young person between the ages of 10 and 24 who uses a cell phone

### Participant recruitment

Participant recruitment will take place in youth centres, school settings, and youth clinics where 15-24 year olds frequent. Trained program staff will recruit participants in sites comprised of both urban and peri-urban, and in-school and out-of-school populations. Younger participants will be recruited primarily from youth centres and secondary school settings, while older participants will be recruited primarily from youth centres as well as technical and university settings. Maternal health clinics where pregnant and parenting young people visit will also be included as recruitment sites. Staff will make brief presentations about the ARMADILLO program during times when youth frequent the recruitment sites. Staff also will place ARMADILLO posters and/or fliers in the targeted youth settings to garner interest. Interested youth will be directed to approach data collectors, who will tell them more about the program and can screen them for eligibility if interested. As part of an exploration of the applicability of this mobile phone program, reasons for interested youth participants being declared *ineligible* to participate (i.e. lack of literacy, lack of mobile phone) will also be noted.

Adults/caregivers will be primarily recruited by program staff and posters placed in markets and shopping plazas near the youth recruitment sites. The morning of the scheduled research activity, staff will phone the participants to remind them of the day’s session.

### Participant consent

Prior to beginning FGDs and pilot testing, staff will obtain informed consent or assent from each participant. As part of the consent process, participants will be given more detailed information about the program’s background, goals, and objectives. They will be told that FGDs may last up to two hours and that piloting will require two visits of 1.5 h as well as a three week period where they will be encouraged to share the ARMADILLO program with their peers. Participants will be told that participation is free, voluntary, and confidential. All FGDs and piloting sessions will be digitally recorded for later review. Participants will be asked to sign an informed consent or assent form but told that their names will not be linked to any of the information they provide, or written on any other form. Participants who complete the discussions and pilot testing will be told that they will be reimbursed for travel costs.

### Consenting minors: parental consent and minor assent

In Phase 1, as part of recruitment of 15-17 year olds, the research team will make every reasonable effort to gain parental consent of these potential participants, while still protecting their privacy and autonomy. The research team will call parents when possible, or else send written information about the study when appropriate. Participants of FGDs will be asked to only provide feedback on ARMADILLO message content, the usability of the system, and youth cell phone use in general. At no point will participants be asked to reveal any information about their personal SRH attitudes and beliefs, nor will they be asked about their own sexual activity.

Due to the minimal risk to participants in Phase 1, as ensured by the generalized, non-personal nature of the FGD content described above, in the event that a parent or appropriate guardian of a minor aged 15-17 cannot be located in order to provide consent, this will not necessarily preclude the minor from joining. The research team respects that, in its attempt to recruit a representative sample of all youth aged 15-17 who may have need of the system, the team will need to recruit from settings or from among youth demographics where identifying and locating a parent/guardian may not be possible. For example, strategies to recruit out-of school youth (including 15-17 year olds) include identifying youth in places where young people gather (plazas, sports fields, markets, etc.). In these settings, identifying a parent or guardian may be especially challenging, and a requirement for parental consent could preclude these groups from participating.

In the event that a parent or guardian cannot be located following a reasonable effort by the research team, the research team will gain consent from the minor and provide the minor with written information on the study, including a number to reach the research team, should the participant or their parents/guardians later have questions. Also, should a minor qualify as an emancipated minor (as legally defined by the study site country), they too will be able to provide their own consent. Recruitment from in-school settings will rely on the assistance of school administrators to facilitate contact with parents and guardians of minors to gain consent. Extensive community outreach in all study sites will ensure that communities are fully aware of the nature of the study being conducted.

Phase 2 pilot and pre-post testing, by contrast, will ask young people to respond to questions about their own sexual attitudes and behaviours, and to intensively use ARMADILLO for a short time period. Due to the more sensitive nature of the research content in this second phase, all youth participants 15-17 years old will be required to obtain parental consent from a parent or caregiver prior to participation. In the case that parental or caregiver consent cannot be obtained, these minors will be considered ineligible for study participation.

In both phases, participants between the ages of 18 and 24, emancipated minors, and adult participants will be required to provide informed consent.

While the names of participants will be recorded on the individual consent and assent forms, they will not be written on any FGD or pilot testing forms. In this way, the anonymity of participants will be preserved while still obtaining documentation of their consent to participate in the adaptation plan.

### Sampling and allocation

Table 1 illustrates the groups of young people that will be involved as participants for each phase of the study and the kinds of sampling that will be used to recruit them. Note that the number of participants indicated in the table is the maximum number of data collection events. The ‘younger’ and ‘older’ youth remain flexible in this core protocol (with only minimum and maximum ages, respectively, established) in order to allow the protocol to be adapted appropriately to each study site based on local definitions and realities of youth and the transitions to adulthood.

**Table 1:** Study Sample Size and Sampling Method

**Phase 1**	**Method**	**Participant**
	Focus Group Discussions (3)	Younger female youth (age determined by site, 15 years-old minimum)
	Focus Group Discussions (3)	Younger male youth (age determined by site, 15 years-old minimum)
	Focus Group Discussions (3)	Older female youth (age determined by site, 24 years-old maximum)
	Focus Group Discussions (3)	Older male youth (age determined by site, 24 years-old maximum)
	Focus Group Discussions (2)	Parents/caregivers and healthcare providers of young people (aged 10-24)
**Phase 2**	**Method**	**Participant**
	Pre-post survey In-Depth Interviews(10)	Younger female youth (age determined by site, 15 years-old minimum)
	Pre-post survey In-Depth Interviews(10)	Younger male youth (age determined by site, 15 years-old minimum)
	Pre-post survey In-Depth Interviews(10)	Older female youth (age determined by site, 24 years-old maximum)
	Pre-post survey In-Depth Interviews(10)	Older male youth (age determined by site, 24 years-old maximum)

### Sample size calculation

This formative, qualitative research study relies on the recruitment and participation of youth and parents/caregivers of youth found in each study site. Each focus group shall have no more than 10 participants, in order to keep the groups small enough so that each participant can fully engage in the discussion. This results in a maximum of 30 younger youth females, 30 younger youth males, 30 older youth females, 30 older youth males, and 20 parents/caregivers of youth participating in Phase 1. Based on ARMADILLO partners’ experience in research and development of similar m4RH content in countries such as Rwanda and Tanzania, this number of FGDs and participants is sufficient to achieve data saturation, a point at which similar themes and reactions are consistently learned across data collection events and very little new information is learned from additional participants.

Phase 2 will have up to 40 youth recruited to participate in pre-post surveys testing various SRH measures and share their own and their peers’ experiences with the platform in an in-depth interview. This number of young people will ensure sufficient reactions to the prototype testing as well as information to inform measure development for the usage and impact stage (Stage 2) of the ARMADILLO study.

### Study instruments

Focus Group Discussion and In-Depth Interview guides are semi-structured and all of the instruments are available upon request.

*Tool 1: Determining eligibility for young people’s participation*: this tool will assess the text message health literacy of young people being screened for participation in Phases 1 and 2.

### Phase 1

*Tool 2: Focus Group Discussion guide for young people:* questions will centre on mobile phone usage and the provision of feedback on ARMADILLO, including: program promotion material; program welcome screen; message content; general impressions; a youth friendly services database; and other formats for communication.

*Tool 3: Focus Group Discussion guide for parents/caregivers of young people:* questions will centre on mobile phone usage among youth and the provision of parent/caregiver feedback on ARMADILLO, including: program promotion material; program welcome screen; message content; general impressions; a youth friendly services database; and other formats for communication.

### Phase 2

*Tool 4: In-Depth Interview for young people: usability guide*: to be used as a mechanism for a member of the study team to walk a Phase 2 participant through the ARMADILLO system, ensuring that participants are confident in their ability access the information they want.

*Tool 5: In-Depth Interview for young people: peer reaction guide*: to be used at a follow-up meeting with Phase 2 participants, this tool consists of a series of questions addressing peer reactions to the ARMADILLO platform and peer feedback around the platform’s usability.

*Tool 6: Pre-post survey for measures testing with young people*: to be implemented at the initial and follow-up meeting with Phase 2 participants, this tool consists of a series of measures on SRH knowledge, acceptability, communication, and self-efficacy, sexual intention, care-seeking behaviour, and pregnancy. These measures are tested in this formative phase for use in Stage 2.

### Analysis

All FGD and interview data will be transcribed in the local languages and then translated into English verbatim and transferred into electronic files containing one transcript for each data collection event. The FGD and pilot testing facilitators will use these digital files and interview notes taken during data collection to write summary “topline” reports of study results. Reports will contain information on common themes about the acceptability, usability, and peer opinions of the ARMADILLO program for young people, as well as actionable steps for program content revision and promotion prior to launch of the main study. Topline reporting is an efficient method to achieve the formative research objective of developing a platform that resonates with youth and aligns with local policies, norms, and guidelines. Findings will be immediately incorporated into message revisions and ARMADILLO program development.

Results from the pre- and post-test surveys will be analysed using descriptive statistics, with results presented separately for the pre- and post-test surveys. The small sample size will permit a basic assessment of the measurement characteristics of each item. Means, standard deviations, and minimum and maximum values will be reported for continuous variables, while percentages will be reported for categorical variables. Change on measures from pre- to post-test will be evaluated quantitatively to investigate whether there was movement on indicators during the 30-day pilot testing period. In addition, correlations across items may be analysed to obtain information about possible relationships among variables.

### Ethical considerations

There will be no risk to those who choose not to participate in the research study. For those who voluntarily consent to participate in the study, because the formative research focuses on sexual and reproductive health information needs and measures as well as the acceptability and appropriateness of the proposed ARMADILLO platform, it is possible that there may be some questions asked which make the respondents feel uncomfortable. Participants will be informed as part of the consent or assent process that they can choose to ignore questions or leave the study altogether at any point if they feel uncomfortable, without repercussion.

Data collectors for focus group discussions, in-depth interviews, and administers of pre- and post-pilot surveys will be the same sex as participants, in order to minimize any discomfort participants may have discussing the subject matter. Participants will be encouraged to identify an environment in which they are comfortable speaking with the research team, and at a time that suits them.

Participants (youth/parents/caregivers/care providers) in Phase 1 and Phase 2 will be reimbursed for travel costs at a local rate within reasonable limits. Participants may be provided with refreshments during the data collection. Additionally, participants may be provided with prototype posters or advertising materials (pins, pens, etc.) from the ARMADILLO programme campaign. Based on conversations with our local partners, travel reimbursement and refreshment provision - rather than participant compensation - is appropriate in this research context, considering what is being asked of the participants.

Participants may stand to benefit from engaging with the study, for example, by learning information about youth sexual and reproductive health. Results from m4RH pilots conducted by partner FHI360 in East Africa suggest that young people’s sexual and reproductive health knowledge improved as a result of using the program. Furthermore, young people reported behaviour change and increased partner and peer communication.

With regards to the study methods themselves, all information collected will remain confidential with the study team.

### Ethics approval

The WHO HRP Review Panel on Research Projects (RP2), comprised of a committee of external reviewers, reviewed and approved the scientific and technical content of the study (protocol ID, A65892). We then obtained ethics review and approval from the WHO Research Ethics Review Committee (ERC).

### Study timeline

The formative research will take approximately twelve months. The initial two months will involve preparatory activities, including introductory planning meetings, convening relevant stakeholder groups, including Message Development Committees, and engaging relevant officials. After this period, approximately two months will be spent by the implementing partner to develop and refine draft messages with the input of the stakeholders group and ARMADILLO partners as needed. Phases 1 and 2 are anticipated to take seven months, with the ARMADILLO platform finalized in the final month.

## Discussion

### Expected study outcomes

As the study will be situated in multiple sites, the results will be applicable to understanding the adaptation process of mHealth technologies for youth across different health and cultural settings. In order to create a platform that is widely accessible, we are developing the platform using SMS technology, taking advantage of a base channel of mobile communication that is available across all mobile phones. This formative stage will feed into the development of later study stages which will rigorously test the coverage and impact of the platform. The outcomes of the study will also inform approaches for scale-up and identify opportunities for integrating the platform into a variety of existing youth-friendly health service resources. Most crucially, the information generated by this study will drive the refinement and customization of a technological tool that will potentially empower youth to adopt positive sexual and reproductive health behaviours.

Beyond the development of the intervention itself, sustained youth participation in this study will capture a comprehensive understanding of the SRH needs and priorities of the youth community. Through FGDs with adult caregivers/parents of youth, this study will also gain valuable input on the sexual and reproductive health needs of youth as perceived by this important group of stakeholders.

### How individuals (male and female youth) are affected by the public health need that the study will address

This study is structured around the development of a tool that is intended to give youth discrete access to tailored, age-appropriate sexual and reproductive health information and resources. This formative study presents the proposed platform and method of delivery to youth males and females in equal numbers, and incorporates their expressed SRH information needs (based on Phase 1 focus group discussions) into the tool so that the resulting prototype is a direct and localized adaptation of youth needs in the study sites. Youth are also the foundation of the resulting piloting phase, ensuring that by the end of this study, when the content is finalized and ready for larger scale deployment and assessment, the end product will be a direct reflection of the needs of the population affected by this study.

### Contribution of the study to identifying and/or reducing inequities in sexual and reproductive health care

This study is structured around improving access to quality SRH information for youth, a population often disenfranchised from traditional sexual and reproductive health interventions. Age, marital status, and financial, legal, and/or cultural sensitivities often result in this population being overlooked by health care providers and policymakers alike. This study focuses specifically in addressing the needs of this population of both males and females, by providing SRH information in a discrete manner, addressing some of the barriers to access specific to youth (e.g. privacy, cultural stigma, etc.).

### Measures to ensure inclusive community involvement

Each in-country research partner will lead extensive community outreach efforts in their respective study sites. A key part of the ARMADILLO outreach strategy features the assembly of ARMADILLO Community Advisory Boards (CABs).

CABs will vary by site but should reflect the culture of the community they serve and provide an opportunity for the community to contribute to the development, implementation, and evaluation of research protocol. Members of the CAB will include a variety of key stakeholders as appropriate for each study site. Examples of CAB members might include representatives from government, NGOs, the health sector, community leaders/ advocates, youth groups, and faith-based organizations.

The goals of the ARMADILLO CABs will be to collaborate with the research team on behalf of the community and ensure that research is acceptable, ethical, and relevant to the lives and issues of the community where the study is conducted. The CAB will support the study team in development of broad shared goals around youth health and wellbeing (transcending any particular focus on SRH) which are linked with the study objectives. Communication between CAB members and outreach back to the larger community on study issues will be encouraged.

### Challenges anticipated and proposed solutions

The ARMADILLO text messaging service is an innovative, private, and easily accessible method for providing family planning information to current and potential contraceptive users. There are several limitations to this program, however. First, to use the service you must own or have access to a mobile phone and be familiar with text messaging. To address this limitation, part of the selection criteria for study sites includes the presence of accessible mobile infrastructure and a high penetration of mobile phones among the population. Second, the service provides cursory information on contraception and the user must access a different source for in-depth information. However, it is worth noting that the short ARMADILLO messages are specially crafted to address the common questions surrounding the subject of interest. Additionally the system will not provide information on services that are not confirmed as being legal and available in the study setting.

With regard to this formative stage of research, there are some challenges anticipated with regards to the process of obtaining consent for the participation of youth, especially at-risk youth who still qualify as minors (15-17). The views of this younger population are critical to capture in this formative stage, in order to ensure that the ARMADILLO messages developed are appropriate and accessible to this slightly younger audience. The consent procedures explained in detail in the Methods section are the result of extended discussion by the study team, implementing partners, and members of the WHO Ethics Review Committee.

Also discussed in detail with partners was the issue of what, if any, risks exist to ARMADILLO study participants in Phase 2 of research. At a glance, the main potential risk to participants is privacy-related, in that an outside person (parent/caregiver, partner, friend) may view the messages on the client’s phone against their wishes. Privacy concerns will be closely monitored for throughout the entirety of this research study, and the research team feels that most potential concerns can be mitigated by getting informed buy-in from a variety of stakeholders in the research site (parents and community leaders serving on advisory groups, in addition to youth themselves). Additionally, participants will also be asked about these possible privacy concerns in focus group discussions. It should be noted, in learnings from developments of similar mHealth programs, that this has proven a non-issue in the past, mostly because communities view mobile phones as a valuable means of providing SRH information to young people and mobile phone users are adept at managing their text messages to maintain sufficient privacy in their communications.

### Plans for dissemination of research findings

Project results will be disseminated in the form of publications that can inform the larger audience of stakeholders this field. Country-specific results will also be shared with key informants and the healthcare community in order to plan for eventual widespread deployment and integration into the health system.

The project is critical for localizing an mHealth platform that can be deployed for widespread use across numerous health systems. The results will generate standard operating procedures and best practices for customizing mHealth strategies, and potentially serve as a global guidance tool for other innovations. The output from this project will also inform subsequent rigorous assessments that will evaluate the coverage and health impact of the tool.

## Footnotes

1 Defined as health-related reading fluency and vocabulary when applied to a mobile, text-messaging platform. Participants will be asked to read several ARMADILLO messages and literacy will be judged by fluency in reading the messages. Text message health literacy will be assessed rather than standard health literacy because previous m4RH work has shown that individuals with low literacy may still text message and be able to comprehend health messages on their mobile phones
